# How tight are beetle hugs? Attachment in mating leaf beetles

**DOI:** 10.1098/rsos.171108

**Published:** 2017-09-13

**Authors:** Dagmar Voigt, Alexey Tsipenyuk, Michael Varenberg

**Affiliations:** 1 Institute for Botany, Technische Universität Dresden, 01062 Dresden, Germany; 2 Department of Mechanical Engineering, Technion—Israel Institute of Technology, Technion City, 32000 Haifa, Israel; 3 George W. Woodruff School of Mechanical Engineering, Georgia Institute of Technology, 801 Ferst Drive, Atlanta, GA 30332, USA

**Keywords:** adhesive setae, attachment, copulation, discoid, sexual dimorphism, sexual selection

## Abstract

Similar to other leaf beetles, rosemary beetles *Chrysolina americana* exhibit a distinct sexual dimorphism in tarsal attachment setae. Setal discoid terminals occur only in males, and they have been previously associated with a long-term attachment to the female's back (elytra) during copulation and mate guarding. For the first time, we studied living males and females holding to female's elytra. Pull-off force measurements with a custom-made tribometer featuring a self-aligning sample holder confirmed stronger attachment to female elytra compared with glass in both males and females; corresponding to 45 and 30 times the body weight, respectively. In line with previous studies, males generated significantly higher forces than females on convex elytra and flat glass, 1.2 times and 6.8 times, respectively. Convex substrates like elytra seem to improve the attachment ability of rosemary beetles, because they can hold more strongly due to favourable shear angles of legs, tarsi and adhesive setae. A self-aligning sample holder is found to be suitable for running force measurement tests with living biological samples.

## 1. Introduction

Sexual selection is one of the agents that drive evolution, influencing biodiversity and survival of species [1]. Female choice often favours conspicuous secondary sex traits in males [2]. Size dimorphism, coloration, visual, acoustic and chemical signals are well-known features facilitating sexual selection in the animal kingdom [1]. In leaf beetles (Coleoptera, Chrysomelidae), numerous species exhibit a distinct sexual dimorphism in tarsal adhesive setae [3]. Typical ‘mushroom-shaped’ setae with discoid terminal elements are commonly found only in males, which are known to attach more strongly than females to smooth surfaces [4–7]. These setae have been assumed to be adapted to hold onto the surface of the female's covering wing (elytra) during copulation and long-term mate guarding [3,7]. Male setae inspired the development of effective biomimetic mushroom-shaped adhesive microstructures [8], which were reported to adhere passively to flat, smooth substrates [9]. However, the assumed adaptation of the male-specific adhesive setae to the attachment to female's elytra is not yet experimentally confirmed. This is caused by challenging execution and interpretation of experiments under nearly natural conditions. Furthermore, beetle attachment is highly complex, because six legs act in concert at four hierarchical levels (axially symmetric legs, tarsi, tarsomeres and adhesive setae). When natural counterparts like female elytra and male tarsal adhesive setae meet each other, two irregular surfaces having different physical and chemical properties form a contact. Numerous studies have been carried out during the past decades in order to understand the mechanisms of insect attachment. These studies considered single leg, tarsus and seta, as well as living beetles on artificial flat and fixed, natural plant leaf surfaces [3,10–17]. As previously suggested, the performance of single adhesive pads and legs is less efficient in isolated state, when compared with the contribution of all legs acting in concert [18–20]. One may conjecture a differing performance of free-acting beetles under the field conditions compared with those in delimited laboratory studies.

The focused attachment mechanism that remains not fully understood is associated with the beetle interaction during mating and maintaining the copulation posture. The smooth or structured surface of female elytra has been thought to be selective for male-specific setae. Strongly adhering male beetles are expected to be more successful competitors and mates. A first approach to this problem was made with water beetles (Coleoptera, Dytiscidae) by using a balance [21]. During copulation the male water beetle attached to the female's plane back surface by specific, sucker-shaped organs on three tarsomeres of each leg, resulting in species-specific pull-off forces between 24.0 mN in *Hydaticus transversalis* Pontoppidanto and 288.2 mN in *Dytiscus marginalis* L. [21,22]. A hundred years later, force measurements in natural conditions at the organism level are still challenging.

In the light of the above, our study focused on the forces required to separate a male leaf beetle from the female elytra. To allow for proper measurements, we used a custom-made tribometer featuring a self-aligning sample holder, which has been successfully applied in the past for studies of synthetic surfaces, including beetle-inspired adhesives [23–25]. This self-aligning holder allowed males to act freely, without influencing the alignment of their legs and tarsomeres. Thus, they could be studied for the first time in operation on the female's back. For comparison, females were pulled off from female's elytra, and both sexes were tested on glass. We were interested to answer the following questions. (1) Is the measurement device applicable to living biological samples? (2) Do males generate higher forces than females on female elytra, while excluding the interlocking of claws? Subsequently, can the present data confirm the assumed adaptation of the male-specific adhesive setae to the female's elytra? (3) Do relatively free-acting organisms in contact with natural, curved counterparts produce different forces than organisms in contact with artificial flat counterparts (e.g. on force platforms or in centrifugal force tests)?

## 2. Material and methods

### 2.1. Beetles

Shiny metallic green–red striped rosemary beetles *Chrysolina americana* L. were used as model organisms. They mainly live in the Mediterranean area, feeding on foliage and shoot tips of rosemary and reach a relatively large body length (5–10 mm) compared with other leaf beetles [26–29]. Hence, they were easy to handle in our approach. *Chrysolina americana* is a representative of the leaf beetle family Chrysomelidae (Coleoptera), to which belong several species used so far in detailed attachment studies (e.g. [3,4,6,11–14,16,17]).

Copulating adult beetles *C. americana* were collected from shrubs of *Rosmarinus officinalis* L. (Lamiaceae) wayside at the Technion campus, Haifa, Israel (32.77°49′89′′ N, 35.02°3′38′′ E), and kept on potted rosemary plants (pot diameter: 22.5 cm, plant height: 40 cm) under laboratory conditions at 24°C, 60% relative humidity, and 14 h photoperiod. Studies with beetles were carried out not later than three days after the field collection.

### 2.2. Microscopy

Supporting information about the beetle attachment system and host substrates was obtained from biological imaging. Ocular and stereomicroscopic observations (Olympus SZX16 with parfocal objectives 0.5 and 1.6, and camera DP26, Olympus Corp., Tokyo, Japan) and cryo-scanning electron microscopy (cryo-SEM, Zeiss Supra 40VP, Carl Zeiss SMT AG, Oberkochen, Germany, combined with the cryo-preparation transfer unit Emitech K1250X, Quorum Technologies Ltd, Kent, UK) on living and freshly dead rosemary leaf beetles were carried out (*N*_♂♂_ = 5, *N*_♀♀_ = 5). For a detailed description of the methods see [30,31]. Footprints of rosemary beetles were visualized according to [32]. Morphometric characteristics of beetle legs were obtained from microscopy images using the image analysis package SigmaScan Pro 5.0.0 software (SPSS Inc.).

### 2.3. Force measurements

Living intact male and female beetles were tested on both flat glass (26 × 5 mm cut pieces of microscope slides, 76 × 26 × 10 mm, Paul Marienfeld GmbH & Co.KG, Lauda-Königshofen, Germany) and convex elytra of a living female. Glass was used as a control surface for comparison with previous laboratory investigations with different leaf beetles.

The glass was cleaned prior to the experiments by successively rinsing with acetone, ethanol and distilled water, and then drying with pressurized air. The roughness values were *R*_a_ = 3.1 ± 0.2 nm for glass and *R*_a_ = 6.5 ± 1.7 µm for the female beetle elytra, obtained over a scan area of 500 × 500 µm; mean ± s.d., *n*_glass_ = 3, *n*_elytra_ = 10 (white light interferometry, Wyko® NT1100, Veeco Instruments Inc., Tucson, AZ, USA). This area roughly corresponds to the average size of tarsomeres in rosemary beetles. The surface free energy was lower on elytra (18.5 ± 1.8 mN m^−1^) than on glass (55.7 ± 0.5 mN m^−1^) (for contact angles and method, see electronic supplementary material, table S1).

Living beetles were weighed prior to the experiments using an analytical balance Ohaus GT410 (capacity: 410 g, readability: 0.001 g; Ohaus Corp., Florham Park, NJ, US).

**Figure 1.**
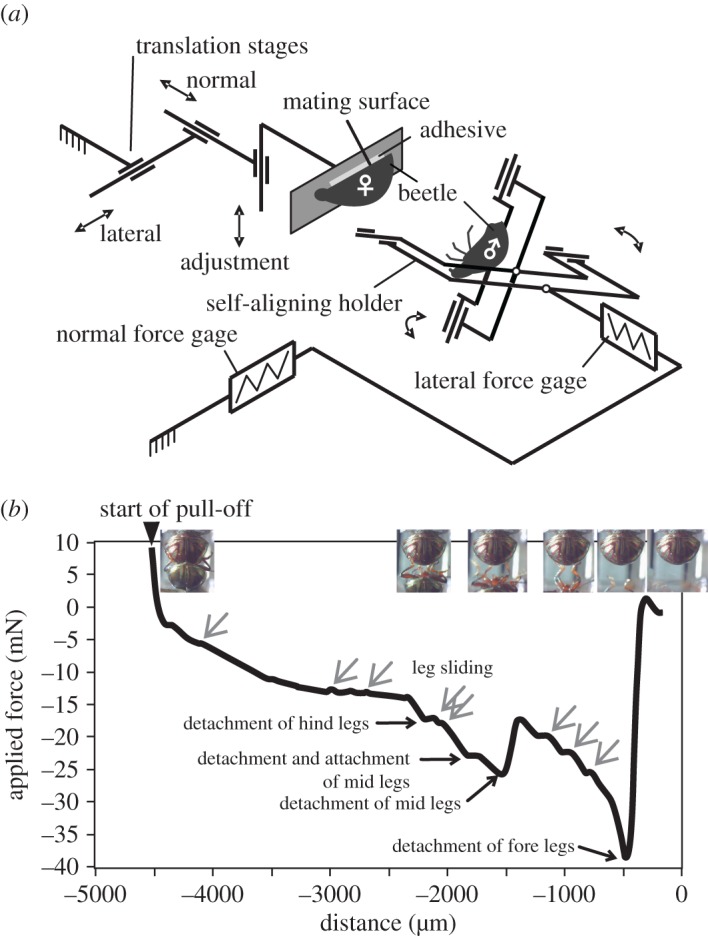
(*a*) Schematic of a tribometer, modified from Murarash and Varenberg [24]. (*b*) A representative force–distance curve obtained for a male in contact with a female's elytra. Distance 0 is defined as the point at which the motion starts and is a relative measurement. Inset images illustrate the posture of the male corresponding to the curve sites below. Grey arrows, pointing diagonally left downwards, indicate the instants of leg sliding.

Forces were measured with the custom-made tribometer incorporating two main units used for driving and measuring purposes (figure 1*a*). The drive unit consisted of two translation stages M-111.1DG (Physik Instrumente, Karlsruhe, Germany) used to load the contact by moving the mating surface in normal and lateral directions, and a linear actuator M-227.10 (Physik Instrumente, Karlsruhe, Germany) used to adjust the position of the mating surface before the experiment. The measurement unit consisted of two pre-calibrated (by known weights) miniature S beam load cells LSB200-50 g (FUTEK Advanced Sensor Technology Inc., Irvine, CA, USA) used to determine the normal (applied) and lateral (friction) forces acting on the sample [24].

Using a mixture of beeswax and colophonium (1:1), test beetles were dorsally attached to a passive self-aligning sample holder based on two orthogonal axes of rotation coplanar with the contact plane [24,25]. Thus, legs and head were freely movable, and the beetle's body could hold a convenient position while contact forces were recorded.

Clean glass (see [33] for the cleaning procedure) and the natural elytra surface of female leaf beetles *C. americana* were used as counter surfaces. To run the tests, glass samples and ventrally fastened living female beetles were attached to the drive unit using the mixture of beeswax and colophonium.

Male and female feet were brought in contact with a female's elytra or with glass at a normal load of 10 mN, kept in contact for 90 s, and then pulled off at a velocity of 100 µm s^−1^ while measuring normal force (figure 1*b*). Experiments were carried out at 25.1 ± 0.6°C, 47.7 ± 2.6%. In total, 100 individual tests were conducted; *N*_♂♂_ = 5, *n* = 15 runs per male, *N*_♀♀_ = 5, *n* = 5 runs per female. Only trials without claw interlocking were taken into account. Fewer runs per female than per male were considered, because females tended to interlock with claws to the edge of glass and elytra due to their larger body and leg span. Thus, less evaluable data were available for females.

Obtained data were evaluated using SigmaPlot 12.0 software (Systat Software GmbH, Erkrath, Germany). Applying *t*-test statistics, both maximum and mean pull-off force as well as safety factor values of individuals were compared between males and females, as well as between elytra and glass. The safety factor was calculated as the ratio of the pull-force over the body weight. In addition, the pull-off force was correlated to the number of shear movements by linear regression.

### 2.4. Video recording

To visualize beetles in contact and during pull-off measurements, videos were recorded using a VEHO VMS-004 Discovery Deluxe USB microscope 40×–800×, and USB uEye® ML (IDS Imaging Development Systems GmbH, Obersulm, Germany); the latter was combined with a Navitar objective (Special Optics Division, Wharton, NJ, USA). In total, 200 video sequences (corresponding to 40 465 still images) were recorded and analysed. Using these videos, we ensured the feet contact with the test substrates and excluded the claw interlocking in trials used for pull-off evaluation. Body postures as well as positions and movements of tarsi and tarsomeres were also analysed based on the obtained video sequences using SigmaScan Pro 5.0.0 software (SPSS Inc.).

## 3. Results

### 3.1. Feet and wing morphology

**Figure 2.**
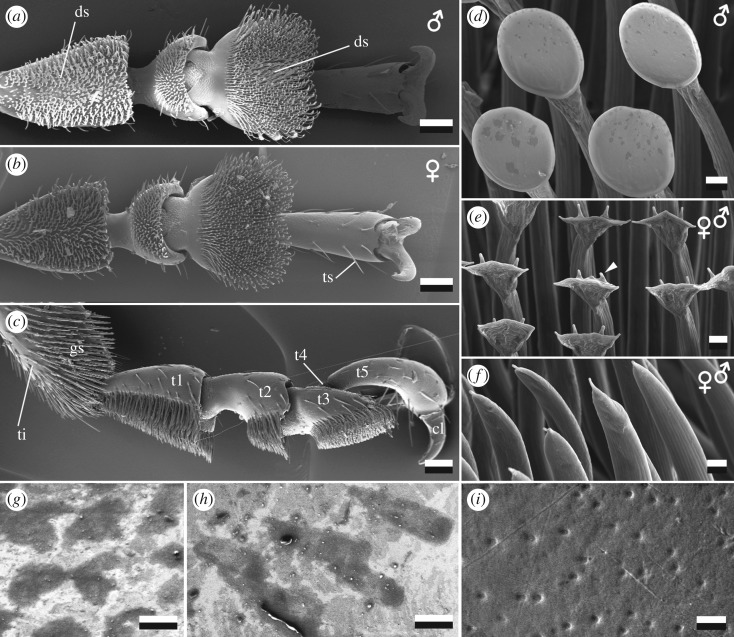
Cryo-SEM micrographs of tarsi of *Chrysolina americana*. Ventral view of male (*a*) and female (*b*) fore-tarsi. Lateral view of female fore-tarsus (*c*), illustrating five tarsal segments (tarsomeres, t1–t5) and distally paired claws (cl), whereas the fourth tarsomere (t4) is hidden. (*d*–*f*) Details of terminal adhesive setae; discoid-shaped ones are present only in males (*d*) and seen as small patches (ds) in (*a*). Spatula-shaped (*e*) and lanceolate-tapered (*f*) terminals occur in males and females. Arrow tip in (*e*) points to approximately 1 µm long setules at the dorsal side of spatula-shaped setae. (*g*,*h*) Footprints left on a palladium-sputtered resin surface after detachment of discoid-shaped setal terminals (*g*) and lanceolate setal terminals (*h*). (*i*) Surface of female elytra showing filled pores and scratches, indicating certain grease layer on the top. ti, tibia; gs, grooming setae; ts, tactile setae. Scale bars: (*a*–*c*) 100 µm, (*d*–*f*,*i*) 2 µm, (*g*, *h*) 5 µm.

The length of fore, mid and hind legs measured 6.2 ± 0.4 mm, 5.3 ± 0.2 mm and 6.1 ± 0.3 mm in males, and 5.8 ± 0.3 mm, 6.2 ± 0.3 mm, 7.0 ± 0.4 mm in females (mean ± s.d., *N*_♂♂_ = 3; *N*_♀♀_ = 3; electronic supplementary material, table S3). The feet (tarsi) of the rosemary beetles were composed of five segments (tarsomeres 1–5) (figure 2*a*–*c*). Tarsomeres were about 444.3 ± 36.4 µm (first), 303.7 ± 18.7 µm (second), 314.6 ± 22.7 µm (third) and 676.1 ± 49.8 µm (fifth) long (mean ± s.d., *n* = 60 per tarsomere; male and female, as well as fore, mid and hind leg pooled together) (electronic supplementary material, table S3). The curved fifth tarsomere bore paired, curved, 226.8 ± 8.2 µm long claws (*n* = 5), having a claw tip diameter of 10.3 ± 2.3 µm (*n* = 10); the diameter of a circle fitting the claw curvature was determined to be 121.6 ± 35.2 µm (*n* = 5) (mean ± s.d., *N*_♂♂_ = 3 and *N*_♀♀_ = 3 pooled together for claw dimensions).

Tarsomeres 1–3 were covered with distally aligned adhesive hairs (setae) having broadened terminals of either discoid (figure 2*d*), spatulate (figure 2*e*) or lanceolate-tapered shape (figure 2*f*). Since we did not detect any sexual differences in claws and non-male-specific tarsal setae in preliminary checks, we pooled data from both males and females for morphometric evaluation. The width was 9.0 ± 0.6 µm in discoid, 8.7 ± 0.5 µm in spatulate and 3.3 ± 0.3 µm in lanceolate-tapered terminals (mean ± s.d., *n* = 20, *N*_♂♂_ = 5 and N_♀♀_ = 5 pooled together for setal dimensions; electronic supplementary material, table S5). The latter were found in both sexes, though in a higher number on female's first tarsomere than on male one (632 and 208 setae, respectively; *n* = 1). The lanceolates' contact area and footprints appeared triangular (figure 2*h*). Spatula-shaped terminals occur only on the third tarsomere in all legs of males and females (514 and 966 setae, respectively; *n* = 1). Male-specific, discoid terminals were found on the first and third tarsomere of the fore, mid and hind legs (figure 2*a*,*d*; 206 versus 98 setae on first and third tarsomere, respectively; *n* = 1). They release nearly circular traces of fluid (figure 2*g*). The terminal disc was surrounded by a meniscus and dorsally conjoined at its lower half with the setal shaft. On single fore legs, the total number of adhesive setae was estimated to be 1236 in males and 1787 in females (*n* = 1 individual, electronic supplementary material, table S2). The mean length of the adhesive setae was quite similar in different setal types, reaching 90.0 ± 9.8 µm in spatulate and discoid and 98.0 ± 8.8 µm in lanceolate (mean ± s.d., *n* = 20). However, setae on the first and second tarsomeres exhibited a distinct gradient of length (figure 2*c*). The length of setae increased from about 70 µm at the base to 150 µm at the distal end. On the third tarsomere, this gradient was less obvious (60–85 µm long setae). All setal shafts were 3.8 ± 0.7 µm in diameter at the base (mean ± s.d., *n* = 20; discoid, spatulate and lanceolate setae pooled).

**Figure 3.**
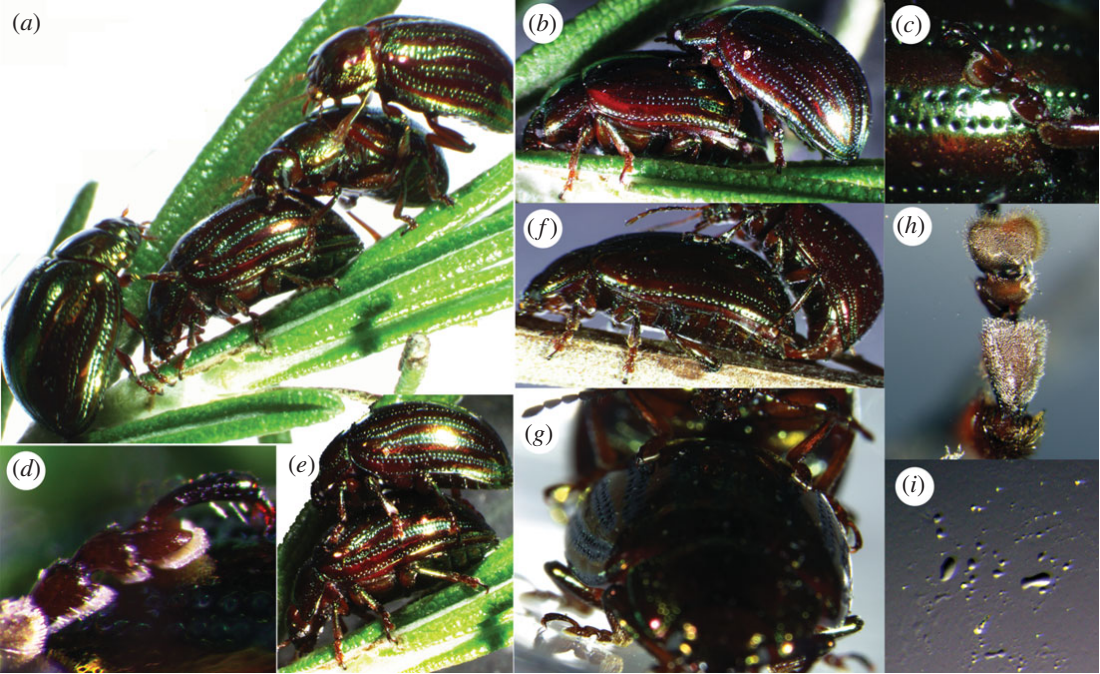
Adult rosemary leaf beetles *Chrysolina americana* (Chrysomelidae) while mating (*a*–*g*), on its host plant *Rosmarinus officinalis* (Lamiaceae) (*a*,*b*,*e*,*f*), and on a glass surface (*g*–*i*). Three males compete for a female, mounting the female and each other (*a*). Optimizing the position before copulation, only several male tarsomeres came in contact with female's back (*b*). Detail of a tarsus in contact with the shiny female elytra surface: all foot segments simultaneously in contact were found only in males firmly attached to elytra (*c*,*d*), but never on the plant surface. Different views of copulation postures (*e*–*g*). Adhesive setae on a male fore tarsus in contact with glass (*h*). Adhesion-mediating tarsal fluid remaining after detachment of the tarsus (*i*).

An elytron was about 7 mm long and 3 mm wide. The diameter of a circle fitting the elytra curvature was 6.2 ± 19.3 mm in cross section and 11.9 ± 0.1 mm in longitudinal section (mean ± s.d., *n* = 2), and the angle between the tangents running from the middle body axis over the left and right flanks of the elytra was 127° (spot check). The surface of the semi-spherical female elytra appeared rather smooth, but it was covered with longitudinally running, paired rows of concave, about 500 nm deep and 37 µm wide pores (spot check; figure 3*c*, diameter) as well as with nano-cones and scratches (figure 2*i*). The edge of the elytra has a pronounced bulge. A circle fitting the bulge curvature measures about 119 µm in diameter (spot check).

### 3.2. Leg positioning and copulation posture

Adult *C. americana* showed various postures of body and legs on the surface of host plants, on elytra of female beetles and on flat glass (figure 3). Because of competition, several males can simultaneously try to mount the female's back, while also climbing each other (figure 3*a*). When successful, a male starts to mount a female laterally backwards. First, it holds on to the elytra with its fore and mid legs, while keeping the hind legs attached to the substrate (figure 3*b*). Subsequently, first three tarsomeres of all legs get in contact with the elytra surface, continuously shearing or short-time pushing for a long period of time. Finally, in copulating, all legs adhere to the elytra, while the hind legs additionally interlock their claws to the margin of the posterior elytra. This position was also observed during the long-term mate guarding after the copulation.

All foot segments were found simultaneously in contact only on elytra and glass (figure 3*c*,*d*,*h*), but never on the host plant surface. On horizontal glass, the beetles hold their feet at a large distance from each other, while the leg segments were oriented at more obtuse angles and the body was kept relatively close to the substrate. However, when attached to the glass in upside-down position, the leg segments were kept at acute angles (electronic supplementary material, Information, Movie S14).

Tarsomeres could be observed continuously moving, mostly shearing, but also shortly pushing with tarsomeres 1–3 in concert (electronic supplementary material, Information, Movie S11). Interestingly, only a portion of the adhesive setae of each tarsomere adhered to the substrate. This portion was continuously switching between different setae. The first and second tarsomeres could be laterally rotated at an angle of 90°, keeping them off the substrate, while the third one remained attached. The first tarsomere was mostly brought in contact with one longitudinal half of the hairy pad. Detachment happened by twisting, short pushing or lateral rolling up of single tarsomeres. Distinct fluid droplets remained on the glass after the tarsal detachment (figure 3*i*).

### 3.3. Attachment

Beetles willingly mounted each other during the experiments. Thus, we expect motivation differences between individuals to be negligible.

**Figure 4.**
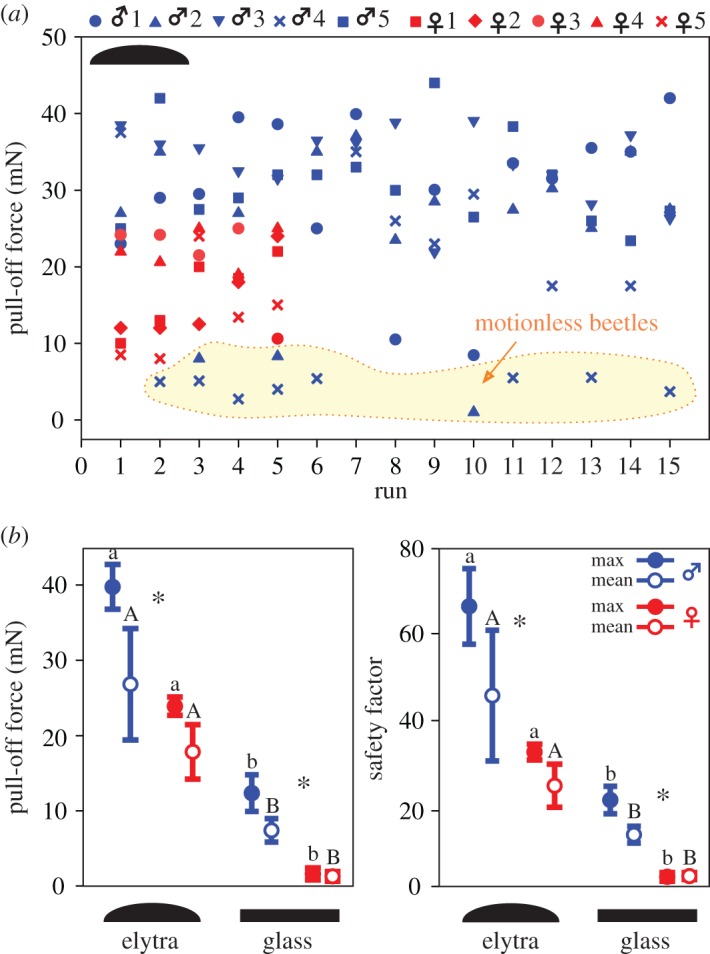
Pull-off forces and safety factors generated by male and female rosemary beetles on female elytra and a flat glass slide. Blue symbols and lines correspond to males and red ones to females. (*a*) Multiple scatterplot of force values for five males and five females, obtained in 15 and 5 consecutive runs on elytra, respectively. The yellow shading, surrounded by the dotted line, indicates values which were obtained by pulling off motionless beetles. (*b*) Scatter plot column, means and error bars, of maximum and mean pull-off forces and safety factors. Asterisks indicate statistical differences between males and females (*n*_♂♂_ = 5, *n*_♀♀_ = 5) according to *t*-test: *t* = 2.4, *p* = 0.041 (mean force), *t* = 11.0, *p* ≤ 0.001 (maximum force), *t* = 2.9, *p* = 0.019 (mean safety factor), *t* = 8.4, *p* ≤ 0.001 (maximum safety factor) on elytra; *t* = 8.4, *p* ≤ 0.001 (mean force), *t* = 9.4, *p* ≤ 0.001 (maximum force), *t* = 11.1, *p *≤ 0.001 (mean safety factor), *t* = 11.9, *p *≤ 0.001 (maximum safety factor) on glass. Different small letters and capitals show differences between elytra and glass for males and females, respectively, according to *t*-test, in males: *t* = −5.7, *p* ≤ 0.001 (mean force), *t* = −16.0, *p* ≤ 0.001 (maximum force), *t* = −4.7, *p* = 0.002 (mean safety factor), *t* = −10.7, *p* ≤ 0.001 (maximum safety factor), in females: *t* = −10.2, *p* ≤ 0.001 (mean force), *t* = −34.3, *p* ≤ 0.001 (maximum force), *t* = −9.6, *p* ≤ 0.001 (mean safety factor), *t* = −30.6, *p* ≤ 0.001 (maximum safety factor).

Pull-off forces ranged from 2.7 to 44 mN on elytra and 0.3 to 15.5 mN on glass, considering both sexes and all individual runs (figure 4). The values varied in the runs per each individual (*n*_♂♂_ = 25, *n*_♀♀_ = 5). However, no distinct trend between forces and the number of runs was detectable (figure 4*a*). The pooling of maximum or mean force values per individual resulted in similar plots and statistics (figure 4*b*). Males adhered significantly better than females on both elytra and glass. Significantly higher pull-off forces must be applied to separate both males and females from elytra compared with glass (figure 4*b*; electronic supplementary material, tables S6, S8).

The body weight was significantly lower in males, 63.5 ± 5.7 mg, compared with that of females, 85.2 ± 7.8 mg (*t*-test, *t* = −2.9, *p* = 0.02, *n* = 5 per sex). Thus, safety factors (force/body weight) were significantly higher in males than in females (figure 4*b*; electronic supplementary material, tables S7–8).

**Figure 5.**
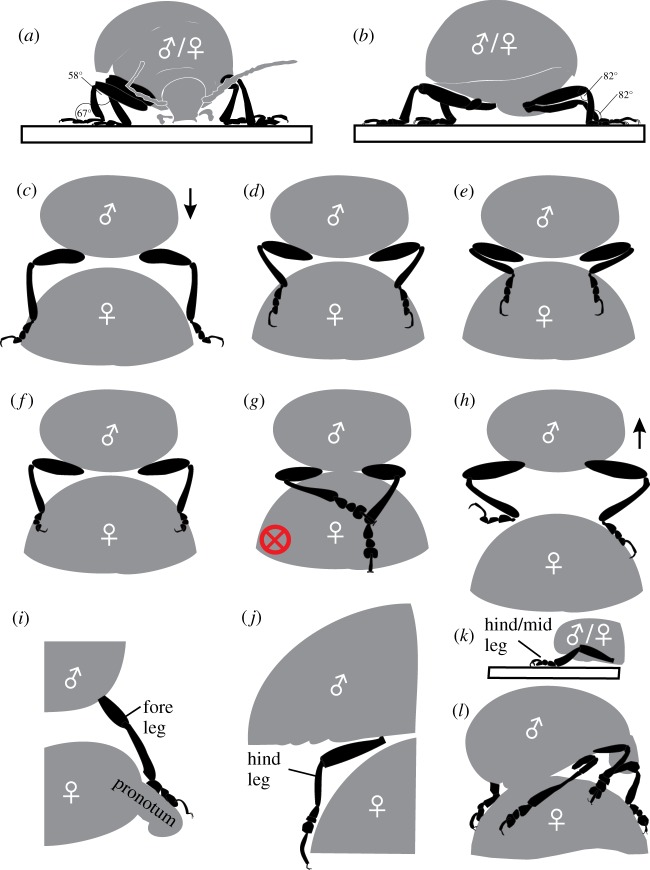
Examples of observed leg postures of rosemary beetles on glass (*a*,*b*) and on female elytra (*c*–*l*). The red cross in *g* indicates an example of a posture (claw interlocking with elytra edge) that was not considered for evaluation. Here, interestingly, the left leg hooked to the right leg with claws, and the claws of the right leg interlock with the elytra edge.

**Figure 6.**
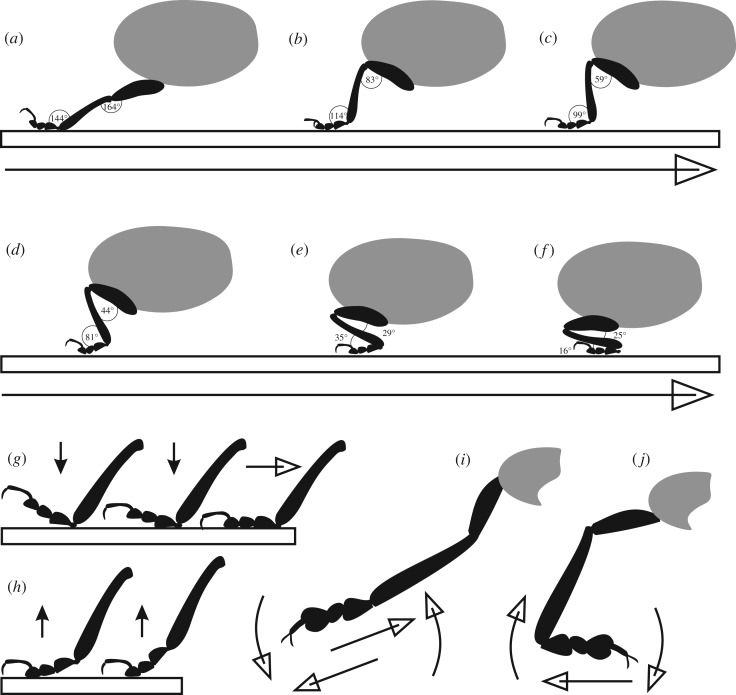
Leg postures of rosemary beetles observed in contact with substrate. (*a*–*f*) A sequence of single-leg motion: extending the leg away from the middle body axis and attachment of the tarsus (*a*), pulling (shearing) the tarsus towards the middle body axis (*b*–*e*) until reaching the most acute angles between the leg segments (*f*). (*g*,*h*) The tarsal attachment and detachment process: regardless of the type of substrate, the proximal tarsomere is brought first in contact with the substrate and is also lifted first for detachment. (*i*,*j*) Movements of the tarsus in the plane of contact: distally aligned tarsus, pulling to the body or pushing away from the body, and moving sidewards back or forward (*i*); 180°-rotated tarsus, sliding away from the body, and moving sidewards back or forward (*j*). Open arrows indicate shearing. Filled arrows point to the direction of lifting and setting the tarsus.

As described in the section ‘Leg positioning and copulation posture’, the postures of beetles differed on glass and elytra; examples are given in figure 5. Moreover, the positions of the legs changed while continuously sliding, attaching and detaching the feet during the contact for 90 s with the substrate; the angles of femur and tibia varied from about 165° to 25° (figure 6; electronic supplementary material, Movies S12–15). Head and antennae were also in motion. Beetles that did not move their feet generated low pull-off forces and were not considered for statistical evaluation (figure 4*a*). The straightened legs of a pulled-out beetle slid against the counter surfaces roughly in parallel to the direction of motion. In most cases, the legs did not detach simultaneously: separation started with hind legs, followed by mid legs and finished with fore legs (figure 1*b*).

In contact with elytra, fore legs were predominantly pulled (sheared) along the longitudinal body axis, finally adhering to the surface of the female pronotum. Mid legs were commonly pulled in the transverse direction to the longitudinal body axis, though they were also observed pushing and pulling laterally. Hind legs displayed repeated pushes and pulls in all directions, including the tarsus rotation at angles of up to 180° (spot check). A single shear event started with the detachment of tarsus and extension of the leg away from the body axis. Then, the tarsus was brought in contact with the substrate, first with the basal tarsomere (figure 6*g*). Owing to this procedure a single male hind tarsus could pull on the elytra at a distance of up to about 2.4 mm (spot check). This value is roughly estimated, because the three-dimensional character of motion made it rather challenging to measure. However, the duration of single shear movements of different legs on elytra and glass as well as a rough number of total shear events per individual during the 90 s of contact in a run could be defined (electronic supplementary material, figures S9, S10). The number of shear events did not influence the pull-off force significantly, as confirmed by linear regressions and *R*^2^ values close to 0 (electronic supplementary material, figure S9). Shear movements in males took longer than those in females (on elytra 5 and 4 s, on glass 7 and 3 s, respectively). Male mid and fore legs on glass pulled the longest period of time (9 s) compared with other runs. Considering the above-mentioned distance, a male hind leg moved at a velocity of about 0.4 mm s^−1^, while being sheared on the elytra surface.

The detachment process started with the lifting of the first tarsomere, followed by the second and third one, while the curved fifth tarsomere with claws acted as a lever (figure 6*h*).

## 4. Discussion

### 4.1. Attachment ability

The tarsal morphology, dimensions, sexual dimorphism and copulation posture of rosemary beetles resemble those of other representatives of Chrysomelidae, e.g. dock leaf beetles, Colorado potato beetles, blue milkweed beetles and Western corn rootworms [3,7,10,14,34–36]. Thus, rosemary beetles *C. americana* are well comparable with leaf beetles investigated in previous studies. The male mounts the female's dorsum, first grasping it with the fore legs, followed by mid and hind legs to get a firm hold. Behavioural observations have elucidated that Colorado potato beetle males may adhere to smooth female elytra during multiple and repeated matings as well as during the long-term mate guarding [37–41]. We suggest a similar performance of rosemary beetles because of the analogue morphology and forces generated on smooth substrates. Differences in the attachment ability between males and females of chrysomelid beetles on smooth substrates have been previously reported [3,5,7,42]. In Colorado potato beetle males, the strong attachment on smooth glass and plastic surfaces was explained by the action of specialized tarsal setae with discoid terminals, presumably adapted to adhere to smooth female elytra during copulation [3,43]. Interestingly, spatulate and discoid terminals in rosemary beetles, exhibit a similar width of 8.7 and 9.0 µm, respectively, resulting in a similar resistance to detachment [44].

Terminal parts of setae are responsible for the generation of friction and adhesion by forming intimate contact with the surface [45]. The authors found a correlation between the friction force, generated by insects on various substrates, and the mean distance between the substrate and setal tips, assuming van der Waals forces to be the only interaction between the surfaces. Besides, the shape of setal tips is differently adapted to different surface roughness [7]. In dock leaf beetles, discoid setae were stiffer in the normal direction (0.7 Nm^−1^) than spatulate (0.4 Nm^−1^) or pointed ones (0.2 Nm^−1^) [43]. This may imply that setal geometry and material property provide different adaptability to smooth and rough surfaces. In fact, softer terminals adjust better to surface irregularities [46], and dock leaf beetle's discoid setae adhered with the highest forces (919 nN), followed by spatulate (582 nN) and pointed ones (127 nN) on glass [43]. The same behaviour is expected for rosemary beetles.

The self-aligning sample holder used in force measurements is found to be well suited to study such living biological samples as mating leaf beetles. Male rosemary beetles generated 1.2 and 6.8 times higher attachment forces as well as 1.5 and 7.9 times higher safety factors on female elytra and glass, respectively. Safety factors obtained with rosemary beetles on glass in the present pull-off experiment are 6.1–38 times lower than those previously generated by Colorado potato beetles and dock leaf beetles on a horizontal, flat smooth resin surface in centrifugal force tests [7,17]. Interestingly, male rosemary beetles performed 6.8 and 7.9 times (force and safety factor, respectively) better than females on glass. This sexual difference is much higher than in Colorado potato beetles (1.1 and 1.3 times) and dock leaf beetles (0.8 and 2.0 times) on flat smooth resin in centrifugal force tests [7,17]. The body weight of females is significantly higher than that of males, partly because of the eggs' load. The attachment force of both males and females of *C. americana* is many times higher than the body weight corresponding to previously reported data for many species with hairy attachment systems, e.g. leaf beetles *Chrysolina polita* L., Chrysomelidae [3].

Pull-off of rosemary beetles from the female elytra resulted in a much better performance (compared with glass) with 2.3 times higher force and 2.9 times higher safety factor in males compared with females. Interestingly, females reliably attached to the female elytra as well, holding up to 34.3 times their own body weight, while lacking male-specific discoid setae presumably adapted for copulation and long-term mate guarding [3,7]. The question arises as to how strongly a male must hold to the female elytra in order to keep attached despite intraspecific competition and female's movements. In this context, safety factors of up to 76.6 in males appear to be more advantageous than in females.

The better attachment performance on elytra could not be explained by the surface free energy of the substrates (18.5 mN m^−1^ on elytra, 55.7 mN m^−1^ on glass) resulting in generally better wettability of glass. However, tarsal adhesion-mediating fluid in insects was considered to behave biphasic, wetting a broad range of surfaces having different chemistry [47–49].

Higher forces measured on elytra can be associated with the direction of contact forces action. On the flat glass, the legs tend to be peeled off the surface (figures 5*a*, 6*h*), so only one tarsomere on each leg can actively resist separation, while on the convex female wings, all tarsomeres can act in concert when pulled in parallel to the elytra surface [50,51]. This is in accord with previous findings that the whole is more than the sum of its parts, i.e. single adhesive pads and legs attach less efficiently when compared with the contribution of all legs acting in concert [18]. In this case, the friction forces, which can be quite high when governed by thin-film-based contact elements [52], can dominate the process. The attachment improves on convex surfaces when the substrate is held between legs, applying higher pressure, as it was recently assumed for climbing frogs and longhorn beetles [53,54]. In addition, convex elytra aligned at an angle of about 127° promote favourable shear angles of legs, tarsi and adhesive setae, and thus, larger friction and higher pull-off forces compared with those on flat glass.

### 4.2. Shear-induced attachment

In contrast to a previous statement that, in Colorado potato beetles, setae with discoid terminal plates do not require shearing for contact formation [7], we observed that, in rosemary beetles, both males and females used shear movements of tarsomeres to get a hold. This supports the model of shear-induced attachment proposed recently [55], as well as observations made with other animals [19,56–59]. The model demonstrates that an applied shear force results in a better alignment of the tarsal spatulae and in an increase of their contact area. On the other hand, shear movements occurred also in male discoid terminals, which is expected to be detrimental for their attachment ability based on previous reports on fibrillar adhesives with mushroom-shaped geometry of terminal tips [60]. The contradiction is made even more pronounced because in our experiments the male beetles generated distinctly lower pull-off forces when they did not shear their feet. One possible reason for this discrepancy may be related to the fact that natural discoid terminals are slanted, with the disc being connected to the seta stem not in the centre, while artificial mushroom-shaped structures are axisymmetric, which presumably makes the biomimetic structures less resistant to shear load. Because males adhered more strongly than females on both flat glass and convex elytra while having less setae on their attachment pads (electronic supplementary material, table S2), we conclude that higher adhesion is generated in discoid than in spatulate terminals. Similarly, dock leaf beetle spatulate setae adhere less than discoid ones, as mentioned above [43].

The proximal (first) tarsomere is set first in contact with the substrate and lifted first for detachment. Interestingly, the highest number of discoid setae occurs on the first tarsomere in males, while only tapered-lanceolate setae are covering it in females. That may be an explanation of the sexual differences in attachment to glass and elytra, assuming the first tarsomere mainly in contact on smooth, flat surfaces like glass. Here, the third tarsomeres and spatulae probably do not attach firmly and thus, do not contribute much to the attachment forces. On the convex substrate (elytra), tarsomeres align to the substrate while sheared due to their morphology and kinematics. As a consequence, the third tarsomere also contacts the surfaces, and pull-off forces increase. Because spatulate terminals are less favourable in attachment to smooth surfaces, pull-off forces in females on elytra are lower than those in males.

Interestingly, males produced the longest tarsal shear movements (on glass), while females' shear movements were shorter. One may conjecture that a proper hold leads to an increase in duration and a decrease in the number of single shear events. However, this consideration is not confirmed by the present study, because there was no significant correlation between the number of shear events and the measured pull-off force.

Since the leg positions greatly vary and continuously change during attachment, we assume that beetle attachment is dynamic rather than static in males and females. Forces measured in natural conditions of copulation posture are lower than those measured previously in other leaf beetles on artificial test substrates by using different test methods, e.g. in centrifugal force tests [7,17] or in experiments with separate tarsal setae and pads [14,15]. Living beetles, walking and holding on natural substrates, probably do not apply their full attachment force. They may tune forces, depending on the situation, by the application of different portions of available setae (shearing) and single tarsomeres (rotation and twisting) (electronic supplementary material, Movie S11).

Shearing, twisting, rotation and lifting movements enable the distinct application of different terminal structures of setae on a single tarsus and tarsomere. Unlike previous statements that distal rather than proximal setae seem to play a more important role in the initiation of adhesion [7], the present study revealed that the proximal setae in tarsomere are brought first in contact with the substrate. Additionally, our observations do not confirm that the tarsus easily buckles or bends by distal or lateral pushing, as it has been previously shown in dock leaf beetles, albeit in a more ‘artificial’ situation than in this study [13]. Rosemary beetles repeatedly pushed their feet, in particular those of the hind and mid legs. While shearing, the tarsi were not always held straight, but frequently at a curved and transverse position, or even rotated at 180°.

Given that the diameter of setal shafts does not vary much (3.8 ± 0.7 µm, mean ± s.d., *n* = 20), shorter setae are stiffer than longer ones. To this end, the distal setae, which are longer than proximal ones, deform easier when brought in contact. This helps in attachment, detachment and shearing that start and finish at proximal tarsomeres.

### 4.3. Assortative mating

The present results exclude the effect of claw interlocking. However, it was repeatedly detected in several runs, in particular when a female attached to a female. Because females are larger than males, their legs span a wider distance, easily touching the wing's edge. Claw interlocking led to a tremendous increase of pull-off force. Such values were not considered in our present evaluations. However, it is worth mentioning that during long-term mating and mate guarding, claws add to a proper hold on female's back. Fore legs are shorter than other legs and grasp the edge of the pronotum, while hind legs are the longest ones and hook to the backmost elytra edge. The circle fitting the claw curvature (diameter of 122 µm) corresponds very well to the circle that fits the elytra edge (diameter of 119 mm). Mid legs usually do not interlock with claws. The male's reliable contact and interlocking with female elytra first depends on the grasping ability of the legs, which is predominantly defined by the leg length. Trochanters and femuri of opposite legs are aligned to span over the elytra width, while tibae and tarsi are held in parallel to clamp the elytra in between them (figure 5*c*–*f*). The total length of male trochanter and femur of 5.2 mm in fore, 4.8 mm in mid and 5.4 mm in hind legs (left and right leg pooled together) matches well the diameter of elytra cross section (6.2 mm). This allows deducing a size-assortative mating as previously reported for Colorado potato beetles [37], whose females have been recorded to choose males according to their size and could show clear preference for individual males [39]. In this context, the surface of the elytra may also provide assortative properties [7]. In rosemary beetles, the female elytra surface appeared smooth despite the presence of a few scratches, pores and nano-cones, which do not provide interlocking sites for claws, considering the claw dimension (figures 2*i*, 3*c*). Moreover, we assume a coverage with epicuticular grease, because pores appear to be filled by a substance which may act as a coupling agent [61]. Thus, physico–chemical interactions between the layer covering the elytra and the adhesive tarsal secretion of the males can be expected. These interactions may be responsible for the formation and maintenance of proper adhesive contact of setae during copulation and mate guarding.

## 5. Conclusion and outlook

The self-aligning sample holder used previously in measuring attachment forces in synthetic samples, allowed representative pull-off measurements with living adult rosemary beetles. Thus, the device is found to be suitable for future studies of biological samples. The strong attachment of males supports previous hypotheses on the adaptation of discoid terminals of tarsal adhesive setae to flat surfaces and long-term attachment during copulation and mate guarding. However, females also generated considerable pull-off forces on elytra, suggesting the ability of spatulate setae to operate well in attachment on convex substrates, including stems and leaves of the rosemary host plant. Beetle attachment is found to be dynamic rather than static. Shear movements of tarsomeres are necessary to get reliable hold in both sexes, i.e. in spatulate and discoid contact elements.

Knowing that mating success often depends on a number of traits [1], several research questions remain open. The future work will focus on the role of claw interlocking, physiological and behavioural influences, physico–chemical interactions at the interface between fluid layers on tarsi and on elytra, different substrate roughness, various natural substrates, external forces generated under such natural conditions as female walking, male competition, etc. and environmental conditions.

## Supplementary Material

Supplementary Information

## Supplementary Material

Supplementary Data_originals

## Data accessibility

The dataset supporting this article has been uploaded as part of the electronic supplementary material.
