# Supplementary material for: How tight are beetle hugs? Attachment in mating leaf beetles
Source: R Soc Open Sci. 2017 Sep 13;4(9):171108. doi: 10.1098/rsos.171108 (PMC5627132; doi:10.1098/rsos.171108)
Supplement: Supplementary Information [file rsos171108supp1.pdf]

Voigt D, Tsipenyuk A, Varenberg M. 2017 How tight are beetle hugs? Attachment in mating leaf beetles. *R. Soc. open sci.* **4**: 171108. http://dx.doi.org/10.1098/rsos.171108

Supplementary Information

Table S1. Surface free energy, its dispersive and polar components (n = 3) as well as contact angles of 1-µl large droplets of three fluids (n = 9 per fluid) obtained on elytra of three female beetles *Chrysolina americana* and on three glass slides, which were used as test substrates in the pull-off experiments (mean ± sd)

	elytra	glass
FSE [mN m^{-1}]	18.5 ± 1.8	55.7 ± 0.5
dispersive component [mN m^{-1}]	14.8 ± 1.5	18.8 ± 1.6
polar component [mN m^{-1}]	3.7 ± 2.8	36.9 ± 2.0
static contact angle		
Aqua Millipore water [°]	95.8 ± 10.0	32.1 ± 2.4
diiodomethane [°]	86.9 ± 4.1	44.2 ± 4.8
ethylene glycol [°]	75.7 ± 3.4	30.2 ± 6.6

See Bräuer et al. 2017 *APIS* **11**, 171–192 (doi:10.1007/s11829-016-9478-0) for the detailed measurement procedure.

Table S2. Number of tarsal adhesive setae having different terminal shape, exemplarily for a male and a female leaf beetle *Chrysolina americana* (n = 1 foreleg)

	discoid	tapered/lanceolate	spatulae	total
male				1236
tarsomere 1	206	208		
tarsomere 2		174		
tarsomere 3	98	36	514	
female				1787
tarsomere 1		632		
tarsomere 2		177		
tarsomere 3		12	966	

Table S3. Leg length in male and female leaf beetles *Chrysolina americana*

length [μm]	male	female
**fore leg total**	**6179.4 ± 351.0**	**5799.2 ± 325.0**
fore leg trochanter	738.9 ± 195.6	805.6 ± 66.0
fore leg femur	1850.6 ± 203.0	1751.3 ± 115.8
fore leg tibia	1749.8 ± 102.0	1602.8 ± 112.8
fore leg tarsus	1840.1 ± 119.8	1639.5 ± 121.8
tarsomere 1	465.3 ± 70.0	397.9 ± 46.3
tarsomere 2	316.4 ± 41.8	285.9 ± 34.2
tarsomere 3	323.6 ± 52.0	309.2 ± 23.9
tarsomere 5	734.8 ± 65.8	646.5 ± 89.8
**mid leg total**	**5286.8 ± 191.2**	**6232.3 ± 249.2**
mid leg trochanter	646.1 ± 96.2	847.2 ± 125.9
mid leg femur	1589.7 ± 109.3	1861.7 ± 119.7
mid leg tibia	1447.1 ± 87.1	1707.2 ± 86.9
mid leg tarsus	1604.0 ± 103.7	1816.2 ± 148.2
tarsomere 1	401.6 ± 52.7	484.5 ± 52.9
tarsomere 2	305.0 ± 36.5	324.8 ± 24.7
tarsomere 3	290.5 ± 43.1	308.9 ± 37.0
tarsomere 5	606.8 ± 65.6	698.1 ± 105.2
**hind leg total**	**6081.1 ± 286.7**	**7022.7 ± 369.5**
hind leg trochanter	941.1 ± 51.9	1206.2 ± 280.6
hind leg femur	1734.4 ± 129.7	2023.8 ± 135.3
hind leg tibia	1737.8 ± 95.2	1934.5 ± 168.8
hind leg tarsus	1667.7 ± 149.3	1858.3 ± 146.4
tarsomere 1	442.2 ± 44.5	468.2 ± 39.7
tarsomere 2	276.6 ± 29.6	313.4 ± 47.5
tarsomere 3	300.0 ± 31.7	355.2 ± 48.8
tarsomere 5	648.9 ± 100.1	721.4 ± 86.5

mean ± sd, $N_{♂♂}$ = 3 pooled together, $N_{♀♀}$ = 3 pooled together, n = 10 per parameter

Table S4. Dimensions of claws of leaf beetles *Chrysolina americana*. Fore, mid, and hind leg, as well as N = 3 ♂♂ and 3 ♀♀ pooled together

	mean	sd	n
claw tip diameter [µm]	10.3	2.3	10
claw curvature diameter [µm]	121.6	35.2	5
claw length [µm]	226.8	8.2	5

sd, standard deviation; n = sample size

Table S5. Dimensions of tarsal adhesive setae having different terminal shape in male and female leaf beetles *Chrysolina americana*. Fore, mid, and hind leg, as well as N = 5 ♂♂ and 5 ♀♀ pooled together; except for discoid: N = 5 ♂♂

	mean	sd	n
spatulate seta's terminal width [µm]	8.7	0.5	20
spatulate seta's length [µm]	90.0	9.8	20
angle of spatulate seta on tarsomer 3	33.7	8.4	10
lanceolate seta's terminal width [µm]	3.3	0.3	20
lanceolate seta'slength [µm]	98.0	8.8	20
discoid seta's terminal width [µm]	8.9	0.6	20
discoid seta's length [µm]	90.0	9.8	20
shaft base width of all seta types [µm]	3.8	0.7	20
shaft width of the tapered part close to the terminal [µm]	1.6	0.6	5
spatules' length [µm]	1.7	0.2	20

sd, standard deviation; n = sample size

Table S6. Pull-off forces [mN] measured in five males (n = 15 per individual) and five females (n = 5 per individual) on female elytra and a glass plate, mean ± sd

♂♂	female elytra	glass plate	♀♀	female elytra	glass plate
1	30.1 ± 10.0	9.3 ± 4.3	1	16.7 ± 5.0	0.7 ± 0.3
2	25.0 ± 10.8	6.5 ± 2.8	2	15.7 ± 5.3	1.0 ± 0.3
3	33.6 ± 5.0	5.4 ± 1.6	3	21.1 ± 6.0	0.8 ± 0.3
4	14.9 ± 12.5	8.6 ± 1.6	4	22.3 ± 2.7	2.3 ± 0.5
5	31.2 ± 6.1	7.5 ± 2.4	5	13.8 ± 6.5	1.0 ± 0.4

Table S7. Safety factors measured in five males (n = 15 per individual) and five females (n = 5 per individual) on female elytra and a glass plate, mean ± sd

♂♂	female elytra	glass plate	♀♀	female elytra	glass plate
1	44.4 ± 14.6	12.1 ± 5.6	1	22.3 ± 6.7	0.9 ± 0.3
2	37.4 ± 16.2	9.7 ± 4.2	2	20.3 ± 6.7	1.3 ± 0.4
3	65.8 ± 9.8	10.6 ± 3.1	3	28.9 ± 8.2	1.1 ± 0.5
4	24.8 ± 20.8	14.3 ± 2.7	4	29.4 ± 3.5	3.0 ± 0.6
5	52.9 ± 10.3	12.7 ± 4.1	5	18.1 ± 8.5	0.5 ± 0.5

Table S8. Pull-off forces [mN] and safety factors pooled for five males (n = 15 per individual) and five females (n = 5 per individual) on female elytra and a glass plate, mean ± sd

	male		female	
substrate	pull-off force	safety factor	pull-off force	safety factor
elytra	27.0 ± 11.3	45.0 ± 20.1	22.4 ± 9.1	29.7 ± 12.2
glass	7.5 ± 3.0	11.9 ± 4.3	1.1 ± 0.7	1.5 ± 0.9

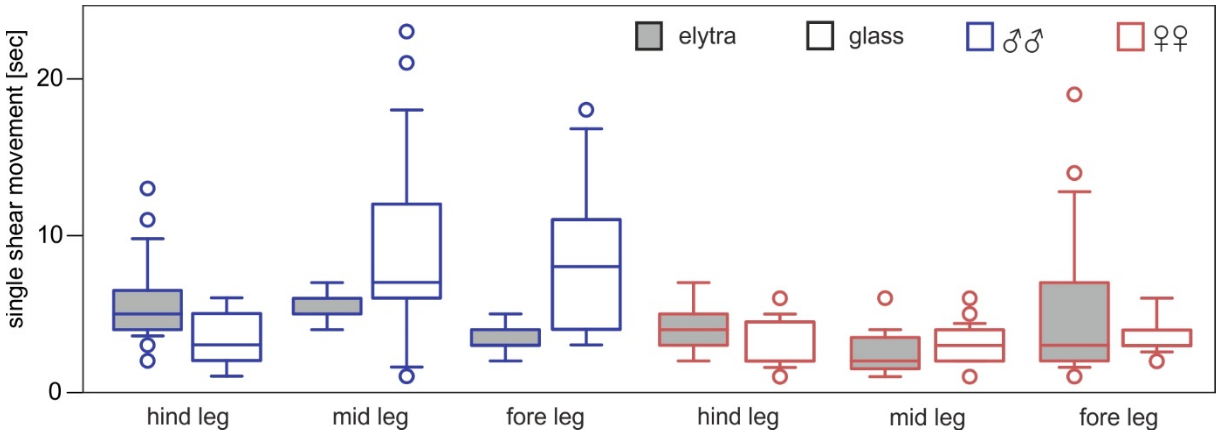

Figure S9. Box plots, representing the duration of shear movements in single legs of males and females (n = 25 counts per leg, surface and sex; 5 individuals per sex pooled together). The ends of the boxes define the 25th and 75th percentiles, with a line at the median and error bars defining the 10th and 90th percentiles; circles are outliers

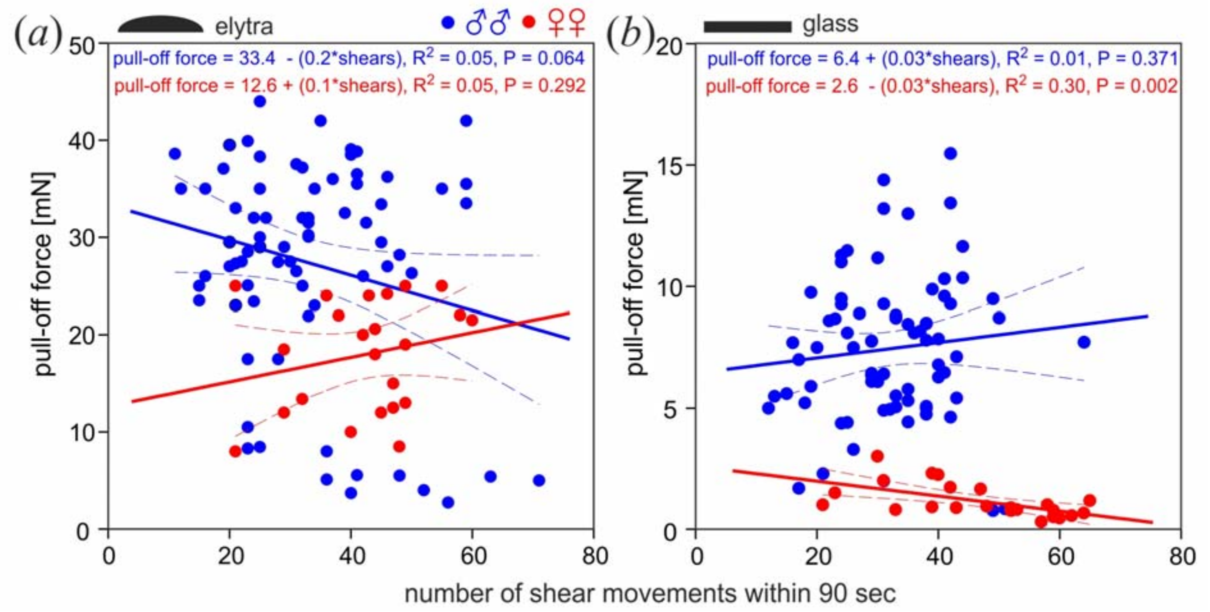

Figure S10. Pull-off force vs number of shear movements within 90 sec on elytra (*a*) and glass (*b*) ($N_{♂♂}$ = 5, $n_{♂♂}$ = 15, $N_{♀♀}$ = 5, $n_{♀♀}$ = 5). The line indicates linear regressions; dashed lines specify the confidence bands at 95% default confidence level; dots are data points, blue: male, red: female

Movie S11. A male set in contact with female elytra, shearing its legs for about 90 s before pull-off test. It can be clearly seen that mid legs are pulled/sheared rather perpendicularly to the body axis, while hind legs pull and push in almost any direction. The chain of hind tarsomeres is curved at times.

Movie S12. A male set in contact with female elytra, shearing its legs for about 90 s before pull-off test. The movements of the right hind leg are shown in detail.

Movie S13. A male set in contact with female elytra, not shearing its legs for about 90 s before pull-off test.

Movie S14. A female set in contact with flat glass, shearing its legs for about 90 s before pull-off test. The movements of the self-aligning sample holder becomes apparent.

Movie S15. A female set in contact with female elytra, distinctly shearing its legs for about 90 s before pull-off test.
